# Acute changes in free and extracellular vesicle-associated circulating miRNAs and myokine profile in professional sky-runners during the Gran Sasso d’Italia vertical run

**DOI:** 10.3389/fmolb.2022.915080

**Published:** 2022-08-26

**Authors:** M. Faraldi, V. Sansoni, S. Perego, M. Gomarasca, L. Gerosa, M. Ponzetti, N. Rucci, G. Banfi, G. Lombardi

**Affiliations:** ^1^ Laboratory of Experimental Biochemistry and Molecular Biology, IRCCS Istituto Ortopedico Galeazzi, Milano, Italy; ^2^ Gruppo Ospedaliero San Donato Foundation, Milano, Italy; ^3^ Department of Biotechnological and Applied Clinical Sciences, University of L’Aquila, L’Aquila, Italy; ^4^ Vita-Salute San Raffaele University, Milano, Italy; ^5^ Department of Athletics, Strength and Conditioning, Poznań University of Physical Education, Poznań, Polska

**Keywords:** physical exercise, circulating extracellular vesicles, circulating microRNAs, myokines, miRNAs target prediction

## Abstract

The modification of gene expression profile, a first step in adaptation to exercise, leads to changes in the level of molecules associated with skeletal muscle activity and energy metabolism—such as myokines—as well as those involved in their transcriptional regulation, like microRNA. This study aimed to investigate the influence of strenuous exercise on circulating microRNAs and their possible association with myokine response. Pre-competition and post-competition plasma samples were collected from 14 male athletes participating in a vertical run (+1,000 m gain, 3,600 m length). Circulating total (t-miRNA) and extracellular vesicle-associated (EV-miRNA) miRNAs were extracted from the pooled plasma. Nanoparticle tracking analysis was performed to investigate pre- and post-competition EV concentration and size distribution. A panel of 179 miRNAs was assayed by qPCR and analyzed by Exiqon GenEx v6 normalized on the global mean. t-miRNA and EV-miRNAs whose level was ≥5-fold up- or down-regulated were validated for each single subject. Target prediction on MirWalk v3.0, Gene-Ontology, and pathway enrichment analysis on Panther v17.0 were performed to define the potential biological role of the identified miRNAs. A panel of 14 myokines was assayed in each sample by a multiplex immunoassay. In whole plasma, five miRNAs were upregulated and two were downregulated; in the EV fraction, five miRNAs were upregulated and three were downregulated. Nanoparticle tracking analysis revealed a similar EV size distribution in pre- and post-competition samples and a decreased concentration in post-competition samples related to pre-competition samples. Gene-Ontology and pathway enrichment analysis revealed that the identified t-miRNAs and EV-miRNAs were potentially involved in metabolism regulation in response to exercise. Correlation between fold-change of the post-competition relative to pre-competition plasma level of both t-miRNAs and EV-miRNAs and myokines further confirmed these results. This study provides an example of a systemic response to acute endurance exercise, in which circulating miRNAs play a pivotal role.

## Introduction

Physical activity (PA) is a part of healthy life, improving its quality and reducing the risk of diseases such as cancer, cardiovascular, metabolic, and cognitive diseases (Lombardi et al., 2016). An entire organism responds to an acute bout of PA by activating an adaptive response that results in the alteration of innumerable biological processes such as angiogenesis, mitochondrial biogenesis, skeletal muscle and bone metabolism, and inflammation (Febbraio, 2017). Although the signaling pathways affected by PA have been defined, the molecular mechanisms that orchestrate the adaptive response still remain poorly understood. Skeletal muscle (SKM) contraction during exercise promotes the release of myokines (Huh, 2018). Besides acting as autocrine and paracrine factors which regulate SKM physiology, the release of myokines into circulation mediate the cross-talk among SKM and other tissues in order to elicit an adaptive response (Pedersen and Febbraio, 2008). Hundreds myokines are expressed and released by SKMs and it has been demonstrated that different kinds of activities regulate their expression differently (Huh, 2018; Gomarasca et al., 2020).

Other circulating molecules, as non-coding RNAs and especially microRNAs (miRNAs), can act as mediators in exercise-induced adaptations (Silva et al., 2017). miRNAs are small non-coding RNAs involved in the regulation of gene expression (Costa et al., 2014; Ha and Kim, 2014). They are considered optimal biomarkers, with diagnostic and prognostic clinical potential in different ambits, since their expression level changes in response to various physiological stimuli (e.g., exercise) and pathological dysfunctions. Furthermore, they are released, and are therefore measurable, in a stable form in almost all bodily fluids (Weber et al., 2010). miRNAs can indeed be secreted into the circulation by virtually all cells: free circulating miRNAs derive from passive release and can be used as biomarkers of cell and tissue response to a stimulus. However, extracellular vesicle (EV)-associated miRNAs instead derive from active secretion and act as paracrine/endocrine factors that promote inter-organ cross-talk (Gyorgy et al., 2011). Due to their suitability in describing pathophysiological responses, changes in the circulating levels of miRNAs may be helpful in depicting physiological adaptation to exercise. PA-associated modifications in circulating miRNAs have been previously assessed (Silva et al., 2017); however, no specific miRNA signatures have been identified that relate to a particular exercise. Few studies have detailed the changes of circulating miRNA levels after a single bout of acute exercise (Baggish et al., 2011; Aoi et al., 2013a; Nielsen et al., 2014; Denham and Prestes, 2016; Barber et al., 2019). Moreover, as myokines, circulating miRNA levels have been reported as sensitive to the kind, intensity, and dose of PA (Sapp et al., 2017).

Recently, EVs have attracted attention in the context of PA; a large number of studies have focused on the changes in EV concentration during PA (Brahmer et al., 2020). Interest in studying EVs derives from their role in cell-to-cell communication (Colombo et al., 2014). Indeed, EVs are able to deliver proteins, nucleic acids, and lipids in a tissue specific manner in order to regulate biological processes, including the systemic PA response (Safdar and Tarnopolsky, 2018). As well as EV concentration, PA has also been shown to affect EV-miRNA cargo (Denham and Spencer, 2020).

PA implies a series of changes in both EVs and free circulating miRNAs; the role of these miRNAs in PA-adaptive response may be better explained in relation to other exercise-associated markers. Therefore, this study aimed to investigate the effects of an acute bout of strenuous exercise on the circulating profile of total miRNAs (t-miRNAs) and EV-associated miRNAs (EV-miRNAs), and their association with the change in myokine levels in non-professional athletes. We thus considered a strenuous form of exercise: a vertical run (+1,000 m gain, 3,600 m length) held in the Gran Sasso d’Italia frame. Understanding the alteration in the circulating profile of miRNAs and myokines could highlight the molecular mechanisms that occur in the adaptative response to exercise.

## Materials and methods

### Study cohort

The study cohort included 14 healthy volunteer male mountain ultra-trail athletes (age: 38.78 ± 10.15; body mass index (BMI) between 20 and 25 kg/m^2^) who were accustomed to high-volume long-endurance exercise performed at vigorous-to-high intensities, as categorized by the American College of Sports Medicine (ACSM) (American College of Sports et al., 2010).

The subjects were sampled 30 min before the competition. Preparation consisted of 8 weeks during which the subjects exercised four to five times per week at intensities of 70%–90% of the maximal heart rate (HRmax) except for warm up, increasing the weekly hours of training from 8 h to 10:30 h. Each training week consisted of the following activities: resistance exercise (80% HRmax); plain running (70%HRmax) and uphill repeated sprints and light running; uphill and/or downhill running (70% HRmax); plain running (80% HRmax) and uphill and/or downhill running (90% HRmax); plain running (80% HRmax) and uphill and/or downhill running (90% HRmax). During the nineth week—the one before the competition—the training volume was halved to allow recovery (Mujika and Padilla, 2003)).

The vertical run consisted of high intensity acute exercise that took place on the Gran Sasso Mountain located in Central Italy. Male runners, from different European countries, competed in a 3.6 km-long run on a 1.03 km vertical ascension with a 29.5% slope. The run is usually completed in 40–50 min at an average speed of 75–80 m/min. During the competition, the weather was fine and the temperature range was 25–27°C.

The study was conducted in accordance with the Declaration of Helsinki. After being informed about all the procedures and the associated potential benefits and eventual risks, all subjects gave their written consent for study participation. The protocol was approved by the ethical committee of Asl Milano 1, Milano, Italia (MARC01), retrospectively registered on 4 January 2018 in (SportMarker, NCT03386981).

### Blood collection and sample preparation

Venous blood samples were collected by antecubital venipuncture in tubes spray-coated with ethylendiaminotetraacetate dipotassium salt (K2EDTA, BD Vacutainer^®^, Becton Dickinson, Franklin Lakes, NJ, United States) in the non-fasted state. Samplings were performed 30 min before and within 30 min after the competition. Blood samples were inverted ten times and then centrifuged, according to the manufacturer’s instructions, at 2000 g, 10 min at room temperature (RT= 22°C) in order to obtain plasma. Plasma aliquots were immediately frozen at −80°C until assayed.

### Nanoparticle tracking analysis

EV concentration and size distribution were analyzed using the NanoSight NS300 system (Malvern Instrument, UK). Three one-minute videos were recorded for each sample. All measurements adhered to the following quality criteria: 20–120 particles per frame, concentration comprising between 10^6^ and 4·10^9^ particles/ml, and amount of valid tracks > 20%. The acquired videos were analyzed by the software provided by the instrument (NanoSight Software NTA). Before measurement, each plasma sample was diluted in an appropriate volume of filtered phosphate buffered saline (PBS).

### Total and extracellular vesicle-associated circulating miRNA profiling

The profiling of t-miRNAs and EV-miRNAs was performed as previously described in Faraldi et al. (2021). Briefly, ice-thawed plasma was centrifuged for 5 min at 3000 g to eliminate cell debris and then pooled together based on the time-point (pre-competition or post-competition).

EVs were isolated from 500 µl plasma using the miRCURY™ Exosome Isolation Kit (Exiqon A/S, Vedbaek, Denmark).

miRNAs were extracted from plasma and EV fraction using the miRCURY™ RNA Isolation Kit (Exiqon A/S). The spike-in UniSp2, UniSp4, UniSp5 were added to each sample at the recommended concentrations of 2.0 fmol/µL, 2.0·10^-2^ fmol/µl, and 2.0·^10–4^ fmol/µl, respectively, in order to test the efficiency of RNA extraction.

Reverse transcription was conducted using a miRCURY LNA™ Universal cDNA synthesis kit II: 4 µl of miRNA-enriched RNA were reverse-transcribed in a total reaction volume of 20 µl (4 µl of 5x Reaction buffer, 9 µl of nuclease free water, 2 µl of Enzyme mix, 1 µl of synthetic spike-in UniSp6 and cel-39-3p). UniSp6 and cel-39 were added at the recommended concentrations of 1.5 10^-1^ fmol/µl and 2.0·10^-3^ fmol/µl, respectively, in order to check for RNA transcription efficiency.

Quantitative polymerase chain reaction (qPCR) was performed using serum/plasma miRCURY LNA™ miRNA focus panel (Exiqon A/S), providing a LNA™ primer set for 179 miRNAs, five RNA Spike-in control primer sets, two blank wells, and six inter-plate calibrators (IPC) (three for each panel). qPCR was carried out on a StepOne Plus instrument (Applied Biosystem, Foster City, CA, United States) using ExiLENT SYBR Green 2X Master Mix (Exiqon A/S) as follows: 10 min at 95°C for polymerase activation and denaturation, 40 amplification cycles consisting of 10 s at 95˚C and 1 min at 60°C, followed by melting curves. Each sample was run in triplicate.

Abnormal amplifications were identified and removed before qPCR data analysis. GenEx software ver6 (Exiqon A/S) was used to analyze miRNA expression, as previously described in Faraldi et al. (Faraldi et al., 2019; Faraldi et al., 2020). The quantification cycle (Cq) of the IPC, provided by the panel, was used to calibrate qPCR plate runs of different experiments. For each sample, only miRNAs with a Cq< 37 were further considered in the analysis. The relative expression level of these miRNAs was obtained by normalizing Cq values on the global mean (Supplementary Table S1) according to Faraldi et al. (2019). An arbitrary value, calculated by GeneEx, was added where data were missed. Three replicates of each sample were averaged after normalization. Any micro hemolysis evidence was excluded throughout the calculation hsa-miR-23a hsa-miR-451a ΔCq (presence of microhemolysis when> 7).

### miRNA validation

EV-miRNAs and t-miRNA which were found ≥ 5-fold up- or down-regulated in pooled plasma were validated in each single sample. EV-miRNAs and t-miRNA were isolated, as described above, from each sample, and reverse-transcribed using the miRCURY LNA™ Universal RT microRNA PCR, polyadenylation and cDNA synthesis kit II (Exiqon). qPCR was carried out on a StepOne Plus instrument, using miRCURY LNA SYBR^®^ Green PCR kit V.5 and pre-designed miRCURY LNA™ microRNA primer set (Exiqon) for hsa-let-7b-3p, hsa-miR-1, hsa-miR-106b-3p, hsa-miR-10b-5p, hsa-miR-127-3p, hsa-miR-128-3p, hsa-miR-133a-3p, hsa-miR-133b, hsa-miR-136-3p, hsa-miR-136-5p, hsa-miR-141-3p, hsa-miR-143-3p, hsa-miR-17-5p, hsa-miR-195-5p, hsa-miR-200a-3p, hsa-miR-205-5p, hsa-miR-210-3p, hsa-miR-22-5p, hsa-miR-29a-3p, hsa-miR-30a-5p, hsa-miR-326, hsa-miR-335-5p, hsa-miR-33a-5p, hsa-miR-34a-5p, hsa-miR-362-3p, hsa-miR-424-5p, hsa-miR-501-3p, hsa-miR-532-3p, hsa-miR-543, hsa-miR-7-1-3p, hsa-miR-874-3p, and hsa-miR-885-5p. The thermal protocol was as following: 10 min at 95°C for polymerase activation and denaturation, 40 amplification cycles consisting of 10 s at 95°C and 1 min at 60°C, followed by melting curves. Each sample was run in triplicate. qPCR were reported as Cq values and were analyzed by the GenEx software ver6 (Exiqon) excluding Cq value ≥ 37. hsa-miR-151a-5p, hsa-miR-30d-5p, and hsa-miR-425-5p were tested as potential normalizers. However, in some samples these miRNAs displayed Cq value≥ 37; they could therefore not be used as reference genes. Hence, global mean was chosen as the appropriate normalization method for qPCR data. The relative expression of each gene was calculated using pre-race samples as control.

### Bioinformatic analysis

An *in silico* analysis was performed to identify the gene targets and the potential molecular pathways regulated by the ± 5-fold (*p* < 0.05) validated EV-miRNAs and t-miRNAs. Target prediction of up- and down-regulated EV-miRNAs and t-miRNAs was conducted in June 2022 using mirWalk ver 3.0 (http://mirwalk.umm.uni-heidelberg.de/) (Sticht et al., 2018), combining three other prediction programs (TargetScan, TarBase, and miRDB). A score of 0.95 was chosen as a threshold. Only target genes listed by at least two tools—always considering TarBase, the database of the experimentally validated miRNA-gene interactions—were taken into account. The number of overlapping target genes among differentially expressed miRNAs was shown by Venn diagram, using Venny (Oliveros, 2007-2015). Gene ontology (GO) enrichment analysis classified target genes into biological process (BP), molecular function (MF), and cellular component (CC). Pathway-enriched analysis on Panther ver17.0 (http://www.pantherdb.org/) (Thomas et al., 2003; Mi et al., 2013; Mi et al., 2019a; Mi et al., 2019b) was performed. Two-sided Fisher’s exact test was used and GO and pathway enrichment *p*-values were corrected, calculating the false discovery rate (FDR). GO categories and pathways were considered significant when the corrected *p*-value was < 0.05.

### Myokine measurement

A panel of 14 myokines known to be released by skeletal muscle during physical activity were assayed through a high sensitivity multiplex bead-based immunofluorescent assay (Myokine Magnetic Bead Panel, Millipore, Burlington, MA, United States) on a MagPix™ Luminex System (Bio-Rad Laboratories, Inc., Hercules, CA, United States) following manufacturer instructions. The following specific myokines were tested in plasma samples: apelin, fractalkine, brain-derived neurotrophic factor (BDNF), osteonectin (SPARC), leukemia-inhibitory factor (LIF), interleukin (IL)-15, myostatin/growth differentiation factor (GDF)8, fatty acids binding protein (FABP)3, follistatin-like protein (FSTL)-1, oncostatin M (OSM), IL-6, fibroblast growth factor (FGF)21, and osteocrin/musclin. Furthermore, plasma irisin concentrations were measured by a competitive enzyme immunoassay (Phoenix Pharmaceuticals, Inc., Burlingame, CA United States), having a sensitivity of 1.29 ng/ml. All samples were tested in duplicate.

### Statistical analysis

Statistical analysis was performed using Prism^®^ v6.01 (GraphPad Software). For extracellular vesicles, myokines and miRNAs, the d'Agostino and Pearson omnibus normality test was performed to analyze data distribution. EV size distribution was analyzed by ordinary two-way ANOVA with Sidak multiple comparison test while pre- and post-competition extracellular concentrations were analyzed using paired parametric *t*-testing. Samples before and after the competition were compared through a parametric paired *t*-test. Pearson correlation analyses were performed to assess relations between changes in t-miRNAs and EV-miRNAs, and those of myokines. Statistical analyses were considered significant when *p* value < 0.05 (*p < 0.05, **p < 0.01, ***p < 0.001).

The power analysis has confirmed the appropriateness of the size of the study cohort (*n* = 14). IL-6 was chosen for the reference parameters due to its responsiveness and key biological role in the metabolic adaptation to exercise, other than a parameter measured in our setting. IL-6 behavior had been assayed in the same cohort in a previous article (Ponzetti et al., 2022). In this setting, a 99% gain in power was made with an effect size of 1.59.

## Results

### Extracellular vesicle analysis

**FIGURE 1:**
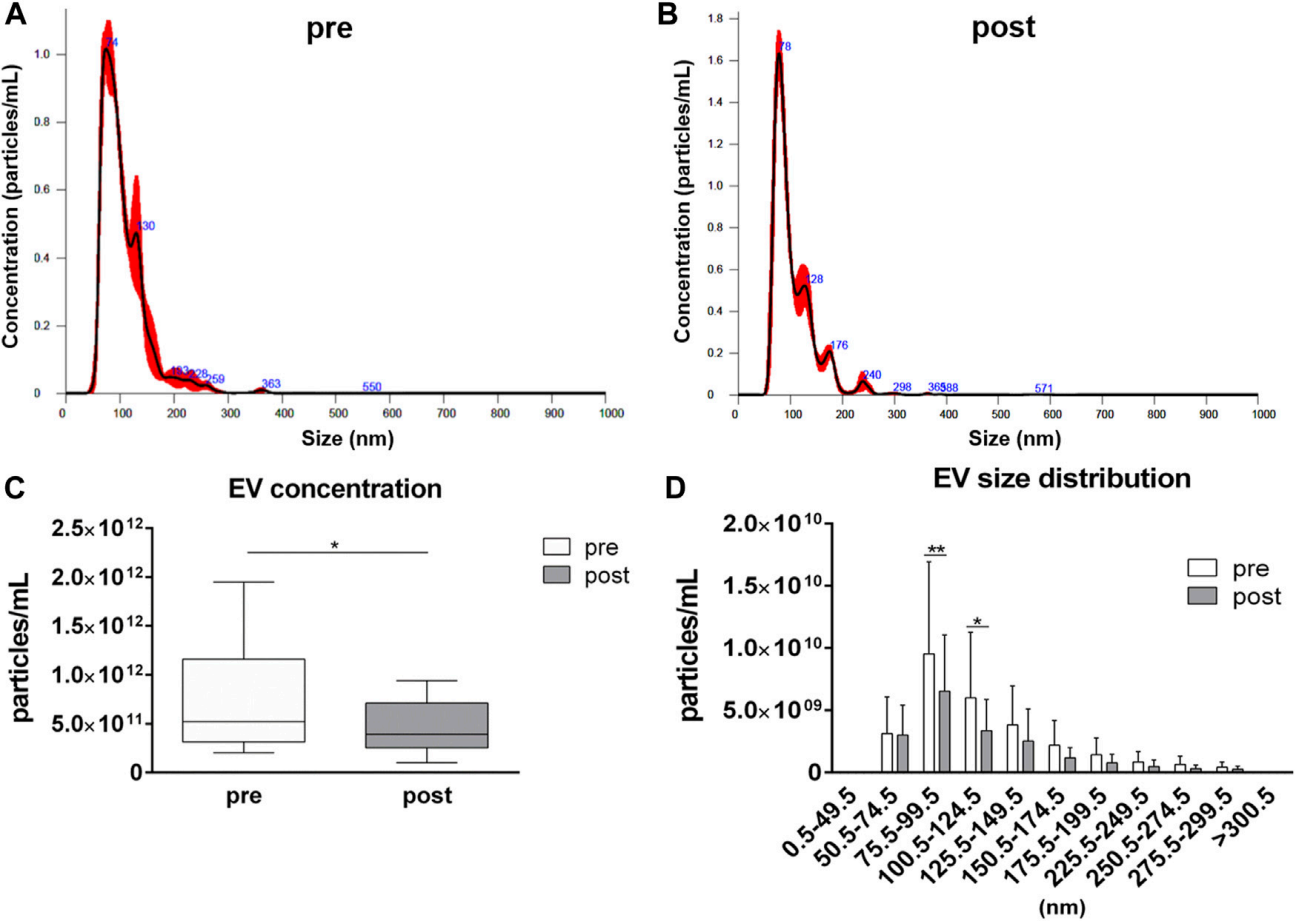
Concentration and size distribution of extracellular vesicles in plasma. Extracellular vesicle analysis for **(A)** pre-competition and **(B)** post-competition plasma samples was performed using Nanosight300. Comparison of total concentration **(C)** and size distribution **(D)** between pre- and post-competition plasma was analyzed. Statistical analysis was performed with Prism® v6.01 (GraphPad Software). Plasma-EV total concentration data are expressed as min. to max. and were compared using a paired *t* test; plasma-EV size distribution data are expressed as mean ± SD and were compared using Sidak’s multiple comparison test. The differences were considered significant when *p* < 0.05. Asterisks indicate significant intergroup differences (*p < 0.05, **p < 0.01, ***p < 0.001).

EV analysis by NTA has revealed similar size distributions in samples collected before and after the competition, with most of the detected particles having a size ranging from 50.5 to 150.5 nm (Figures 1A,B). However, a reduction (*p* = 0.044) in the averaged EV concentration in plasma samples after the competition, related to pre-competition samples, was observed: the average value of particles per mL were 7.71·10^11^ ± 5.20·10^11^ and 4.47·10^11^ ± 2.52·10^11^ in pre-competition and post-competition plasma samples, respectively (Figure 1C). For each EV-size range, EV concentrations in post-competition plasma samples were generally lower than in pre-competition plasma samples, showing significant differences for 75.5≤ diameter< 100.5 nm small EVs (Thery et al., 2018) (pre-competition: 9.53·10^9^ ± 7.39·10^9^ particles/mL; post-competition: 6.52·10^9^ ± 4.52·10^9^ particles/mL; *p* = 0.005) and 100.5≤ diameter< 125.5 nm medium/large EVs (Thery et al., 2018) (pre-competition: 5.99·10^9^ ± 5.26·10^9^; post-competition: 3.36·10^9^ ± 2.50·10^9^; *p* = 0.023) (Figure 1D) (Thery et al., 2018).

### Acute exercise-dependent modulation of circulating t-miRNAs and EV-miRNAs

The circulating level of 179 miRNAs was analyzed in both whole plasma and EV fraction, before and after the vertical run. Eighteen t-miRNAs were ≥5-fold up-regulated and three t-miRNAs were ≥5-fold down-regulated post-competition compared to the pre-competition plasma samples (Supplementary Table S2). Considering the EV fraction, five EV-miRNAs were ≥ 5-fold up-regulated and 11 were down-regulated (Supplementary Table S2). Of these miRNAs, five were modulated in both fractions after the competition: hsa-miR-33a-5p, hsa-miR-34a-5p and hsa-miR-501-3p were up-regulated in whole plasma and down-regulated in the EV fraction, hsa-miR-205-5p was down-regulated in whole plasma and up-regulated in the EV fraction, while hsa-miR-200a-3p was up-regulated in both fractions. Moreover, hsa-miR-208a-3p, hsa-miR-365a-3p, and hsa-miR-497-5p were undetectable in pre- and post-competition plasma samples in both fractions, while hsa-miR-133a-3p, hsa-miR-136-3p, hsa-miR-141-3p, and hsa-miR-338-3p were undetectable only in whole plasma.

### Validation of the identified t-miRNA and EV-miRNAs

**FIGURE 2:**
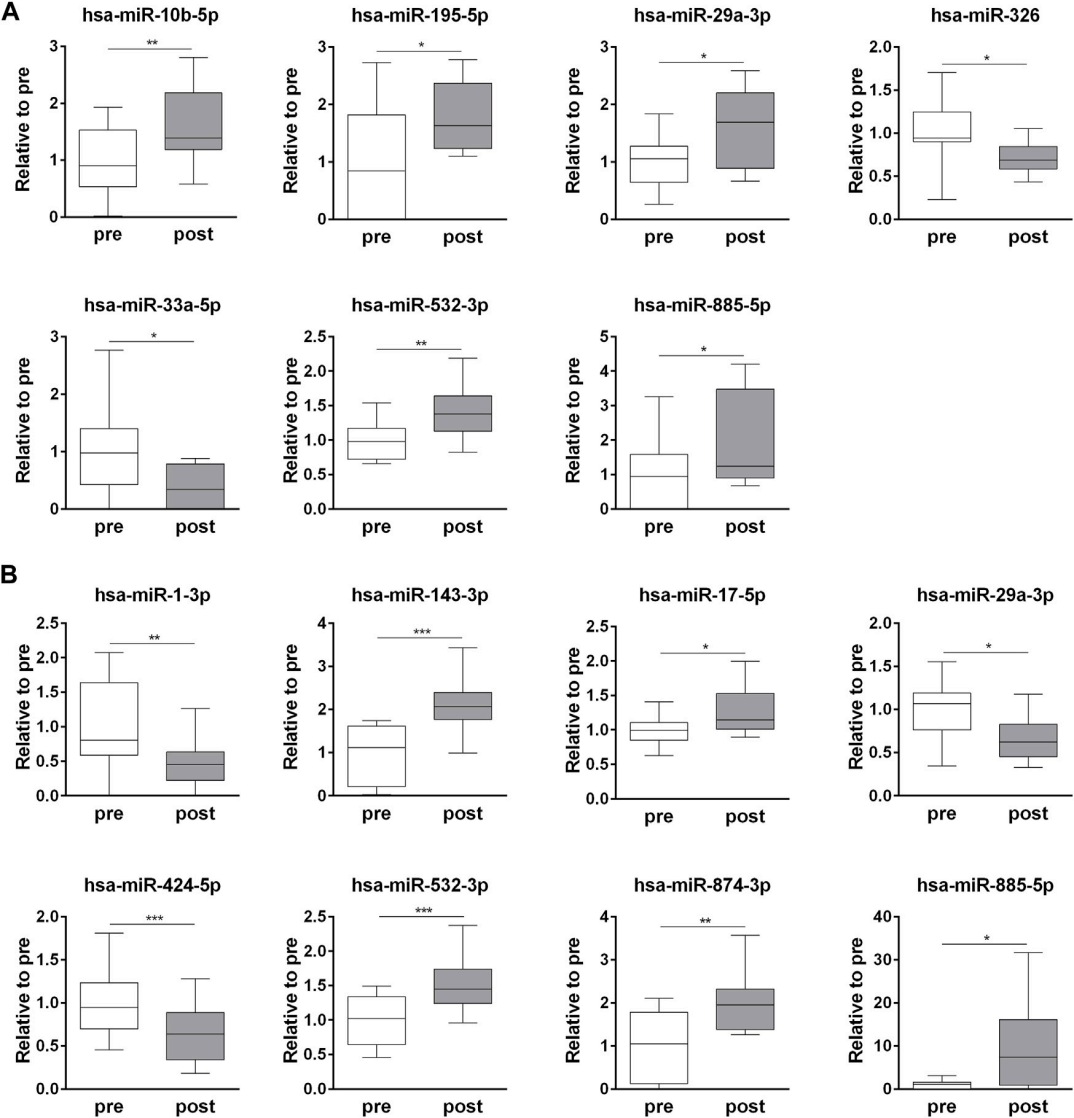
Endurance exercise-dependent changes in circulating plasma t-miRNAs and EV-miRNA. Fold change (post-competition vs. pre- competition) of plasma **(A)** t-miRNAs and **(B)** EV-miRNAs (≥ ± 5 fold, *p* < 0.05), validated in each plasma sample. Statistical analysis was performed with Prism^®^ v6.01 (GraphPad Software). All data are expressed as min. to max. and compared using a paired *t*-test. The differences were considered significant when *p* < 0.05. Asterisks indicate significant intergroup differences (*p < 0.05, **p < 0.01, ***p < 0.001).

The exercise-dependent changes in the circulating levels of all 32 miRNAs, identified in whole plasma and EV fraction, were validated in each subject (Supplementary Table S3). Among the t-miRNAs, five miRNAs were confirmed as up-regulated (*p* <0.05) (hsa-miR-10b-5p, hsa-miR-195-5p, hsa-miR-29a-3p, hsa-miR-532-3p, hsa-miR-885-5p) and two as down-regulated (*p* <0.05) (hsa-miR-326, hsa-miR-33a-5p) (Figure 2A, Supplementary Table S3). Of the EV-miRNAs, five miRNAs (hsa-miR-143-3p, hsa-miR-17-5p, hsa-miR-532-3p, hsa-miR-874-3p, hsa-miR-885-5p) were up-regulated (*p* < 0.05) and three (hsa-miR-1-3p, hsa-miR-29a-3p, hsa-miR-424-5p) were down-regulated (*p* < 0.05) after the competition (Figure 2B, Supplementary Table S3).

### t-miRNA and EV-miRNA target prediction

**FIGURE 3:**
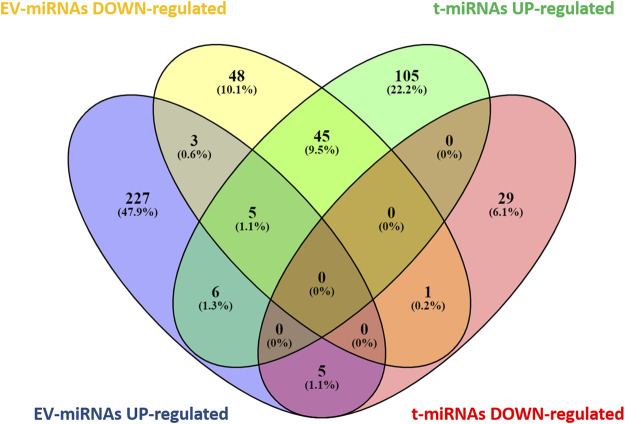
Venn diagram of target genes predicted for t- and EV-miRNA. Graphical representation of common predicted target genes between t- and EV-miRNAs. Venn diagram was based on comparison of the 161 target genes predicted for up-regulated t-miRNAs, the 35 targets predicted for down-regulated t-miRNAs, the 246 targets predicted for up-regulated EV-miRNAs, and the 102 target predicted for down-regulated EV-miRNAs. Venn diagram analysis was performed using Venny [(27) https://bioinfogp.cnb.csic.es/tools/venny/index.html].

Based on target prediction by miRwalk ver 3.0 (last search on June 2022), a total of 196 genes were expected to be targeted by the seven t-miRNAs whose circulating levels were affected by the effort (Figure 3, Supplementary Tables S4, S5) and 348 genes by the eight identified EV-miRNAs (Figure 3, Supplementary Tables S6, S7). More specifically, 161 genes were potential targets of the five up-regulated t-miRNAs and 35 of the two down-regulated t-miRNAs, while 246 genes were predicted as targets of the five up-regulated EV-miRNAs and 102 of the three down-regulated EV-miRNAs (Figure 3). The Venn diagram (Figure 3) revealed that 65 genes were predicted to be targeted by both t-miRNAs and EV-miRNAs.

### Gene ontology enrichment analysis of t- and EV-miRNA targets

**FIGURE 4:**
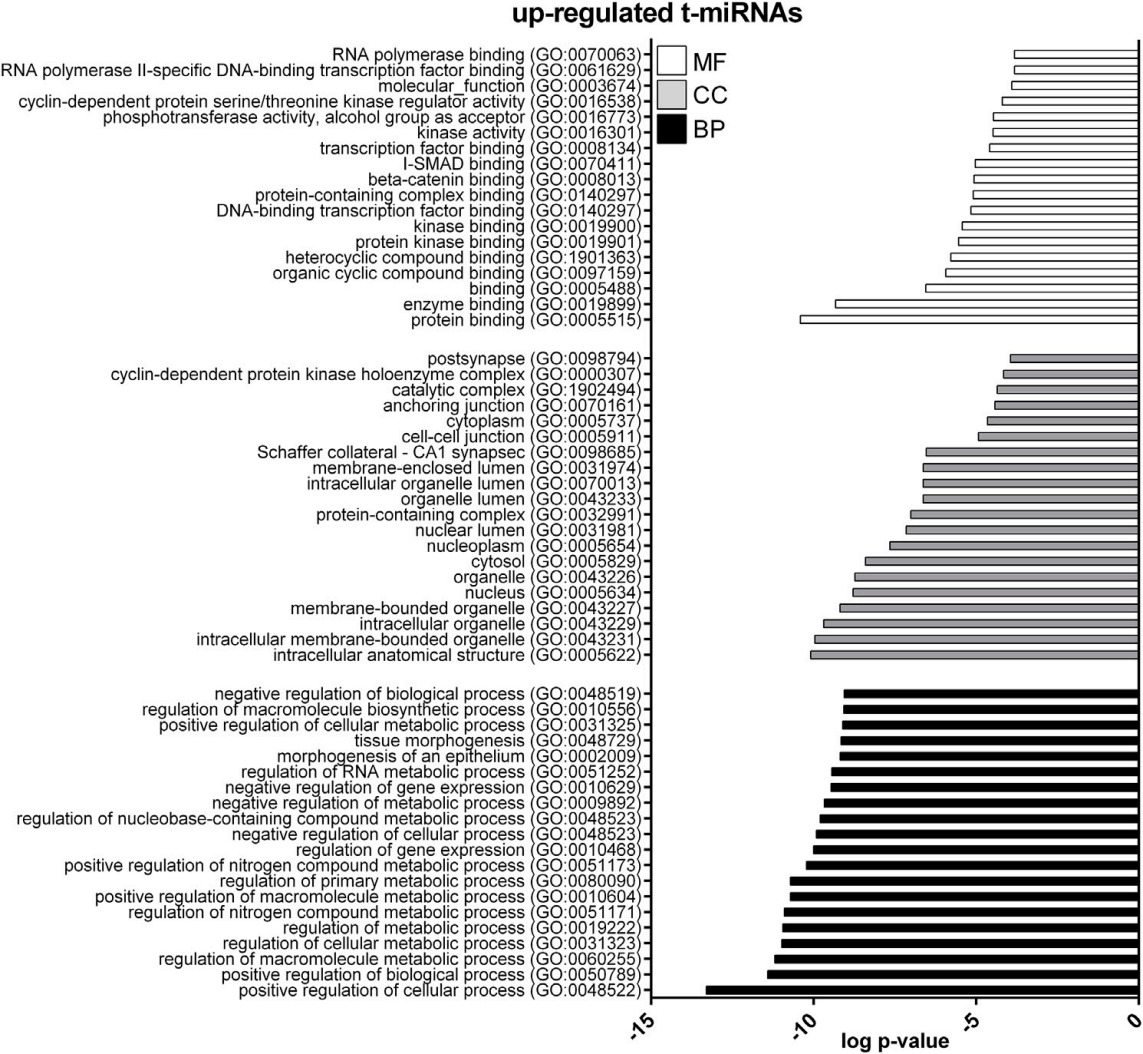
Gene ontology (GO) of predicted target genes for up-regulated t-miRNAs. GO of the 161 target genes predicted for up-regulated t-miRNAs performed with Panther ver 17.0 (http://www.pantherdb.org/). Genes were classified based on biological processes, cellular component, and molecular function. The first 20 most enriched categories were shown. Statistical overrepresentation tests showing the *p*-value of each category are shown in Supplementary Table S7.

**FIGURE 5:**
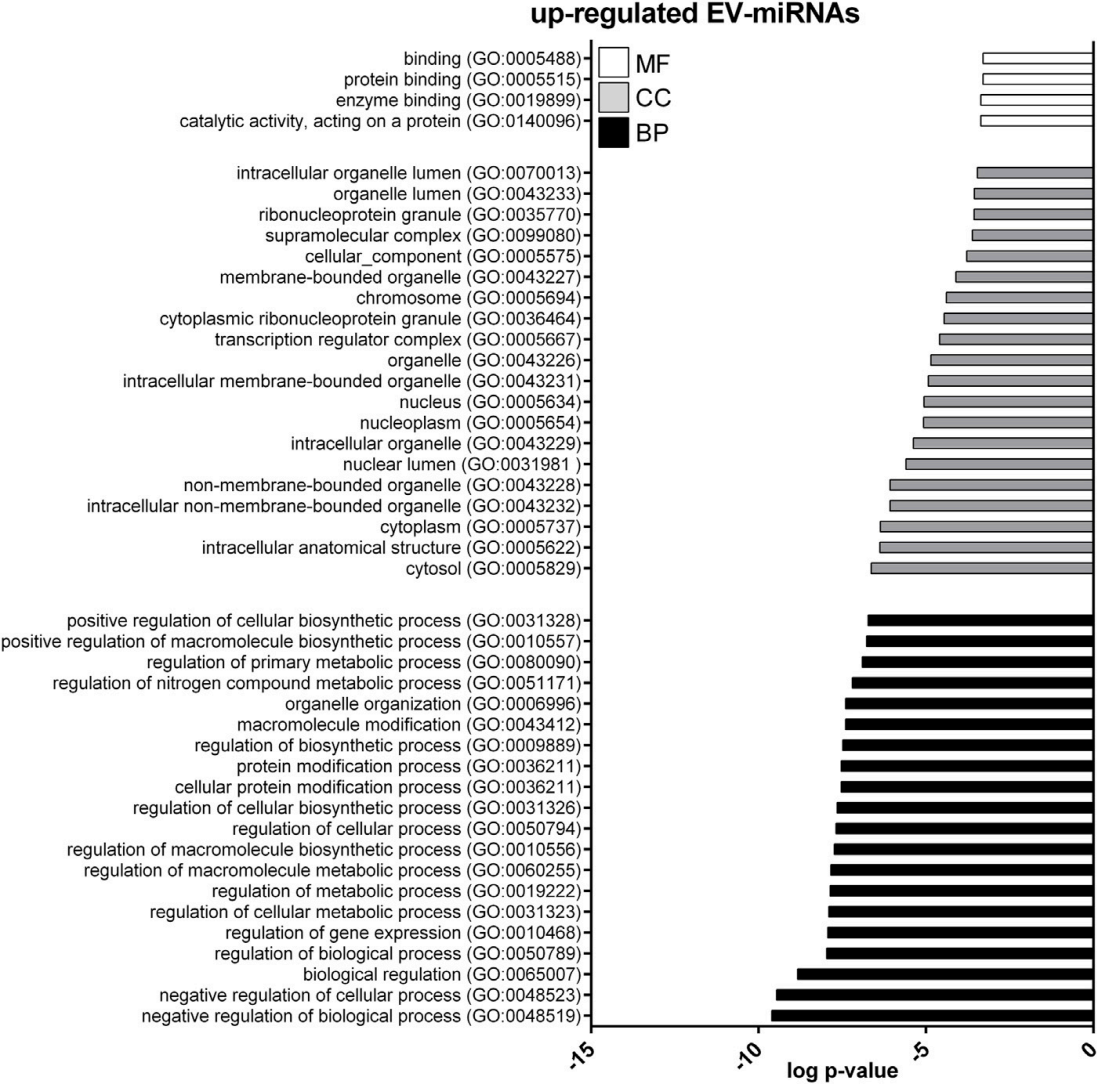
Gene ontology (GO) of predicted target genes for up-regulated EV-miRNAs. GO of 571 target genes predicted for up-regulated EV-miRNAs performed with Panther ver 17.0 (http://www.pantherdb.org/). Genes were classified based on biological processes, cellular component, and molecular function. The first 20 most enriched terms were shown. Statistical overrepresentation tests showing the *p*-value of each category are shown in Supplementary Table S7.

**FIGURE 6:**
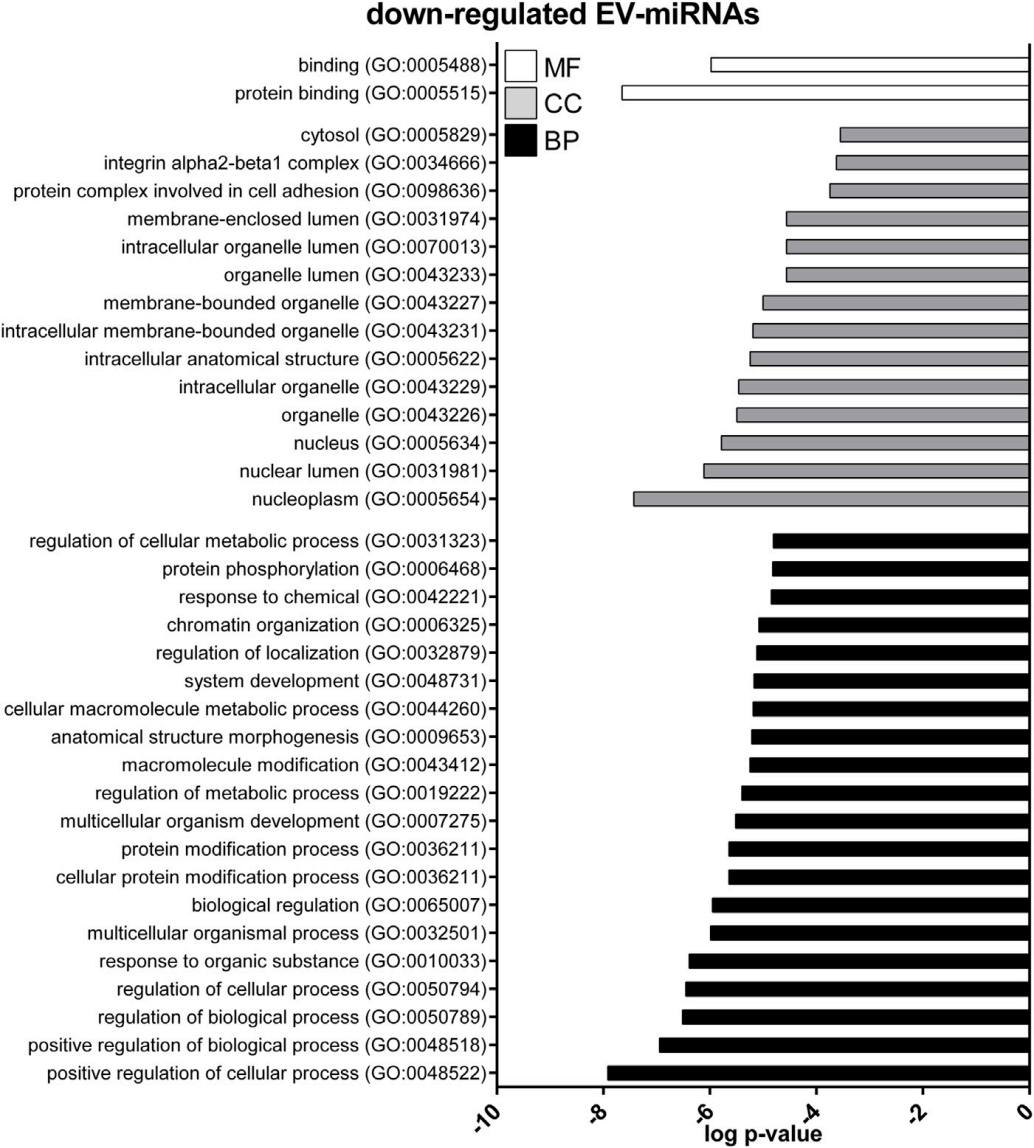
Gene ontology (GO) of predicted target genes for down-regulated EV-miRNAs. GO of 287 target genes predicted for down-regulated EV-miRNAs performed with Panther ver 17.0 (http://www.pantherdb.org/). Genes were classified based on biological processes, cellular component, and molecular function. The first 20 most enriched categories were shown. Statistical overrepresentation tests showing the *p*-value of each category are shown in Supplementary Table S7.

GO enrichment analysis was performed to investigate the biological function of the predicted target genes for t- and EV-miRNAs modulated by exercise using Panther. The top 20 terms for each category, for target genes of both t- and EV-miRNAs were shown in Figures 4–6 and in Supplementary Tables S8–S10. The GO analysis for the target of up-regulated t-miRNAs revealed 455, 40, and 19 enriched terms in BP, CC, and MF, respectively (Figure 4; Supplementary Table S8). No BP, CC, and MF terms were statistically associated with down-regulated t-miRNAs target genes. The GO analysis for targeting up-regulated EV-miRNAs revealed 97, 21, and four enriched terms in BP, CC, and MF, respectively (Figure 5, Supplementary Table S9), while 58, 14 and two enriched terms in BP, CC, and MF, respectively, for down-regulated EV-miRNAs (Figure 6, Supplementary Table S10).

### Pathway enrichment analysis of t-miRNA targets

**FIGURE 7:**
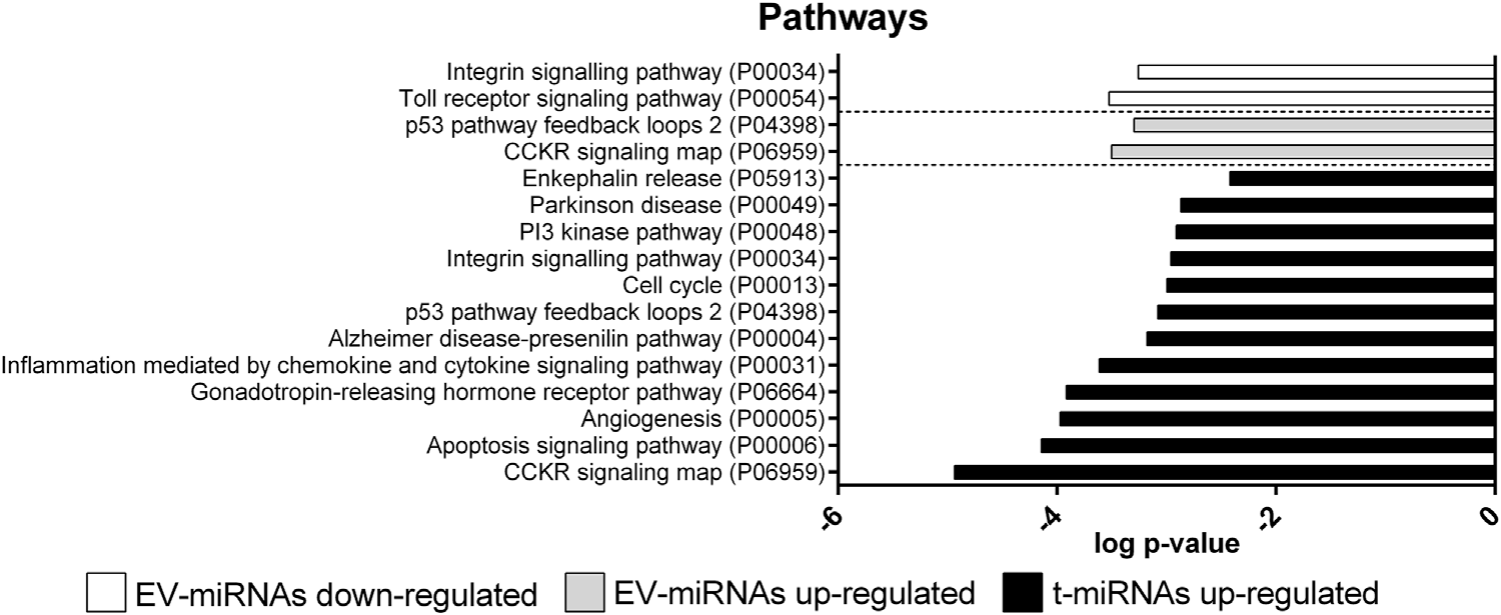
Pathway enrichment analysis of target genes predicted for up- and down-regulated t- and EV-miRNAs. Pathway enrichment analysis for the target genes predicted for up-regulated t-miRNAs (*n* = 161), up-regulated EV-miRNAs (*n* = 246), and down-regulated EV-miRNAs (*n* = 102). Analysis was performed using Panther ver 17.0 (http://www.pantherdb.org/) and all the pathways were shown. Statistical overrepresentation tests showing the *p*-value of each pathway are shown in Supplementary Tables S7–S9

Only 49 target genes predicted for t-miRNAs, the level of which increased by activity, were potentially involved in 12 pathways (Figure 7, Supplementary Table S8): CCKR signaling map (*n* = 9, P06959); gonadotropin-releasing hormone receptor pathway (*n* = 9, P06664); inflammation mediated by chemokine and cytokine signaling pathway (*n* = 8, P00031); angiogenesis (*n* = 8, P00005); integrin-signaling pathway (*n* = 7, P00034); apoptosis-signaling pathway (*n* = 7, P00006); Alzheimer’s disease-presenilin pathway (*n* = 6, P00004); Parkinson’s disease (*n* = 5 P00049); p53 pathway feedback loops 2 (*n* = 4, P04398); PI3 kinase pathway (*n* = 4, P00048); enkephalin release (*n* = 3, P05913); cell cycle (*n* = 3, P00013). No pathways were identified for target genes predicted for t-miRNAs whose level decreased by the activity.

### Pathway enrichment analysis of EV-miRNA targets

Only 13 target genes predicted for EV-miRNAs whose levels were increased by the activity were potentially involved in two pathways: CCKR signaling map (*n* = 9, P06959) and p53 pathway feedback loops 2 (*n* = 5, P04398) (Figure 7, Supplementary Table S9). Nine target genes predicted for EV-miRNAs whose levels were decreased by the activity were potentially involved in two pathways: integrin-signaling pathway (*n* = 6, P00034) and toll receptor-signaling pathway (*n* = 4, P00054), as shown in Figure 7 and Supplementary Table S10.

### Acute exercise-dependent modulation of cytokine plasma level

**FIGURE 8:**
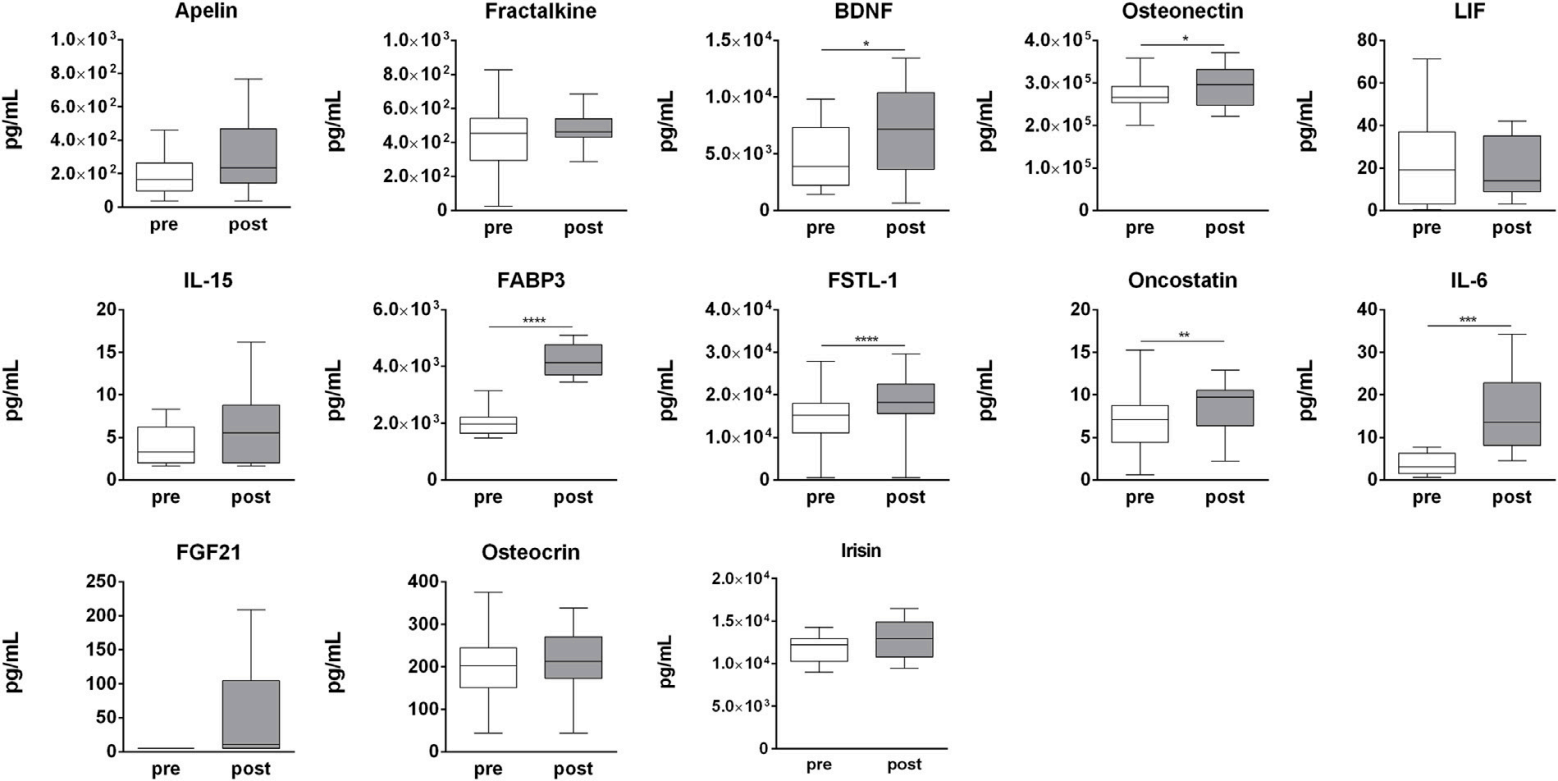
Myokines plasma profile. Changes in myokines profile in pre-competition vs. post-competition samples. Statistical analysis was performed with Prism^®^ v6.01 (GraphPad Software). All data are expressed as min. to max. and compared using a paired *t*-test. The differences were considered significant when *p* < 0.05. Asterisks indicate significant intergroup differences (*p < 0.05, **p < 0.01, ***p < 0.001).

In order to determine the effect of strenuous physical activity on circulating cytokines, a panel of 14 cytokines (apelin, fractalkine, BDNF, SPARC, LIF, IL-15, myostatin, FABP3, FSTL-1, oncostatin M (OSM), IL-6, FGF21, osteocrin, and irisin) was assessed in pre- and post-competition plasma samples. Of these, BDNF (*p* = 0.009), SPARC (*p* = 0.029), FABP-3 (*p* = 0.001), FSTL-1 (*p* = 0.007), OSM (*p* = 0.007), IL-6 (*p* = 0.001), and FGF21 (*p* = 0.031) were significantly up-regulated in post-competition samples compared to pre-competition samples. None of the analyzed cytokines were found to be negatively modulated after the competition (Figure 8). Only myostatin was undetectable at both time-points (data not shown).

### Cytokines and circulating miRNAs

Significant correlations were only considered between validated t-miRNAs and EV-miRNAs and significantly modulated cytokines. Related to the t-miRNAs, results revealed that FABP-3 positively correlated with the levels of hsa-miR-29a-3p (*R* = 0.78, *p* = 0.001) and hsa-miR-885-5p (*R* = 0.72, *p* = 0.004), while negatively with hsa-miR-17-5p (*R* = −0.71, *p* = 0.004); OSM positively correlated with hsa-miR-532-3p (*R* = 0.66, *p* = 0.010). With regard to EV-miRNAs, osteonectin negatively correlated with hsa-miR-33a-5p (*R* = −0.65, *p* = 0.012).

## Discussion

Adaptive response to endurance exercise is a complex phenomenon owing to a multitude of variables. Since myokines and circulating miRNAs (free miRNAs and miRNA encapsulated into EV) have emerged as potential markers of this adaptive response (Safdar and Tarnopolsky, 2018; Domanska-Senderowska et al., 2019), their quantification might provide an understanding of the molecular events that occur during PA. To further investigate such a mechanism, this study sought to analyze the alterations of EV and the modulation of circulating miRNAs and myokines in response to a strenuous bout of endurance exercise. Responsiveness to acute exercise was assayed on a population of 14 habitual ultra-trail male runners who competed in a vertical run (+1,000 m gain, 3,600 m length) held in summertime at Gran Sasso d’Italia.

In the last few years, increasing evidence has demonstrated that circulating miRNA profile is affected by both acute and chronic endurance training (Ryan and Sapp, 2019) in a dose-, intensity- and exercise-type-dependent manner (Ramos et al., 2018). Previous studies were mainly focused on the effect of exercise on circulating miRNAs from total plasma or serum (Ryan and Sapp, 2019); however, in the last few years there has been a growing interest in exercise-induced EV release (Fruhbeis et al., 2015; Guescini et al., 2015). Several studies have shown altered EV concentration after exercise. As observed by Rigamonti et al., 2020, after moderately intense aerobic exercise, the concentration of EVs decreases immediately after an exercise bout. Furthermore, the frequency distribution of EV sizes does not change across the observation (Rigamonti et al., 2020). However, other authors have described an exercise-induced increase of EV concentration immediately after the effort (Fruhbeis et al., 2015; Brahmer et al., 2020). This discrepancy can be explained by the different exercise intensities and durations applied in these settings compared to ours. Notably, also in these studies, the pre-to-post frequency distribution of EV sizes remain unchanged. Our data have also confirmed previous results (Sapp et al., 2017) about the altered regulation of both t- and EV-miRNA signatures in response to acute endurance exercise. In our study, a panel of 179 miRNAs was analyzed in parallel in plasma and the EV fraction. Eighteen and three miRNAs were found, respectively, up- and down-regulated in plasma, whilst five and eleven were found, respectively, up- and down-regulated in EVs in post-competition samples. As well as the different exercise-associated miRNA signatures, the two fractions differed also in miRNA detectability as seven t-miRNAs (hsa-miR-133a-3p, hsa-miR-136-3p, hsa-miR-141-3p, hsa-miR-208a-3p, hsa-miR-338-3p, hsa-miR-365a-3p, and hsa-miR-497-5p) and three EV-miRNAs (208a-3p, hsa-miR-365a-3p, and hsa-miR-497-5p) were undetectable.

According to the validation step, hsa-miR-10b-5p, hsa-miR-195-5p, hsa-miR-29a-3p, hsa-miR-532-3p, and hsa-miR-885-5p resulted in being up-regulated, while hsa-miR-326 and hsa-miR-33a-5p were down-regulated after the competition in plasma; hsa-miR-143-3p, hsa-miR-17-5p, hsa-miR-532-3p, hsa-miR-874-3p, and hsa-miR-885-5p were up-regulated and hsa-miR-1-3p, hsa-miR-29a-3p, and hsa-miR-424-5p were down-regulated in EVs. The observed differences in the miRNA expression profile between plasma and EV fraction, explained by the selective packaging of some miRNAs into EVs rather than their passive release into the bloodstream, were particularly informative about the potential role of miRNAs as biomarkers and on their implications in cell-to-cell communication. This is particularly evident for hsa-miR-29a-3p, which shows an opposite regulation between plasma (up-regulated) and EV (down-regulated) samples.

Moreover, it emerges that not all skeletal muscle-derived miRNAs are affected by endurance exercise; indeed, hsa-miR-1 is the only specific myo-miRNA modulated in our experimental setting and its decrease was observed only in EVs. However, other circulating miRNAs showed a different expression profile in response to the endurance exercise, suggesting that tissues other than SKM are sensitive to exercise in terms of miRNA expression.

To investigate the potential biological functions of modulated miRNAs, bioinformatic analyses were carried out through miRWalk, which combines TargetScan, TarBase, and miRDB database. Target genes with a score ≥0.95 and identified in at least two databases, but always including TarBase, were considered. The majority of target genes were predicted for EV-miRNAs, specifically for up-regulated EV-miRNAs (246 target genes), while 161, 102, and 35 target genes were predicted for up-regulated t-miRNAs, down-regulated EV-miRNAs, and down-regulated t-miRNAs, respectively. The GO analysis identified the predicted targets as mainly involved in metabolic processes. Moreover, considering both t-miRNAs and EV-miRNAs, the enriched molecular function (binding, protein binding, and enzyme binding), and the enriched cellular component, including all cellular compartments (cytoplasm, nucleus, and organelle), suggest a complete enrollment in exercise-induced adaptation at both the molecular and cellular level. Crucial pathways in the response to acute exercise, involved in angiogenesis, cells growth, and inflammation, might be modulated by the miRNAs identified in our experimental setting (Feng and Levine, 2010; Gorski and De Bock, 2019; Gomarasca et al., 2020). The PI3K pathway (Schiaffino and Mammucari, 2011), angiogenesis (Bloor, 2005), p53 pathway (Feng and Levine, 2010), and inflammatory pathways (inflammation mediated by chemokines and cytokine signaling pathway; toll receptor signaling pathway) (Gomarasca et al., 2020) were enriched in t-miRNA and EV-miRNAs predicted target genes. To better understand the relationship between endurance exercise, SKM function, and alteration of circulating miRNA profile, the association between modulated t-miRNA or EV-miRNAs and the blood concentrations of relevant myokines was investigated. To this purpose a panel of 14 myokines was analyzed; six (IL-6, BDNF, osteonectin, FABP3, FSTL-1, oncostatin) were found to be significantly up-regulated after the competition. Our results confirm previous studies on exercise-induced modulation of circulating cytokines (Gomarasca et al., 2020). IL-6 is a pro-inflammatory cytokine expressed in liver and other cell types as vascular endothelial cells, activated monocytes/macrophages, and fibroblasts (Tanaka and Kishimoto, 2014). IL-6 is also expressed in SKM tissue during muscle fiber contraction (Huh, 2018), where it exerts anti-inflammatory functions and enhances energy metabolism, increasing glucose uptake and fatty acid oxidation (Nylen et al., 2018) through the activation of different signaling pathways—including 5'-adenosine monophosphate-activated protein kinase (AMPK) signaling (Kelly et al., 2009). During and after the SKM fiber contraction, IL-6 is also released into circulation; several studies have demonstrated the exercise intensity- and duration-dependent release of IL-6 (Huh, 2018). According to these studies, IL-6 results significantly increased in post–competition samples, as previously tested in our own cohort (Ponzetti et al., 2022). BDNF is a member of the neurotrophin family, mainly expressed in the brain and involved in neuronal cell development (Schnyder and Handschin, 2015). However, it is also expressed in SKM, in response to physical exercise, where it is involved in metabolism regulation (Roh et al., 2017; Lucas Soares Marcucci-Barbosa et al., 2019). *In vitro* experiments have demonstrated the increase of BDNF in SKM cells where it enhances fatty acid oxidation via AMPK after electric stimulation (Matthews et al., 2009). Although both BDNF gene and protein increase have been demonstrated in SKM (Pedersen et al., 2009), it has been estimated that the main source of circulating BDNF during exercise remains the brain (Rasmussen et al., 2009). FABP3 is a small cytoplasmic protein, a member of the fatty acid-binding protein family. It is most abundantly expressed in heart and skeletal muscle, where it promotes the uptake and transport of fatty acids toward mithocondrial β-oxidation system (Furuhashi and Hotamisligil, 2008). Kusudo and colleagues have shown that FABP3 overexpression in C2C12 cells increases AMPK phosphorylation and glucose uptake (Kusudo et al., 2011). Moreover, exercise increases the expression levels of FABP3 in both SKM and in circulation (Lammers et al., 2012; Fortunato et al., 2018). Follistatin is a member of the TGF-β family and is involved in SKM growth by inhibiting myostatin (Wallner et al., 2017). It has been demonstrated by Hansen and colleagues that follistatin levels in plasma increase during exercise; however, the primary source of follistain is not SKM subjected to exercise but the liver (Hansen et al., 2011). OSM is a member of the IL-6/LIF cytokine family that is involved in cell growth and in the angiogenic and inflammatory networks (Richards, 2013). It has been demonstrated that both OSM expression in SKM tissue and serum levels increase immediately after one hour of swimming exercise in mice (Hojman et al., 2011). However, a 10 km run has not affected the plasma level in healthy men immediately after the competition (Lucas Soares Marcucci-Barbosa et al., 2019). Osteonectin is a glycoprotein identified as a structural component of bone, although it was also observed expressed in other tissues (Lane and Sage, 1994). Aoi and colleagues identified SPARC as a cytokine secreted by both human and mice SKM after a single bout of aerobic exercise (Aoi et al., 2013b), although its expression levels were not affected by an acute bout of supramaxial exercise (Norheim et al., 2011; Songsorn et al., 2017). In SKM, SPARC seems to act directly on AMPK to regulate glucose metabolism by increasing solute carrier family 2, facilitating glucose transporter member 4 (GLUT4) expression (Song et al., 2010).

As described above, most myokines modulated in our experimental setting are involved in the activation of the AMPK signaling pathway. AMPK has a central role in metabolic adaptation during energy depletion by inducing fatty acid oxidation and glucose uptake. During stress situations, as exercise, active AMPK induces glycogen breakdown to restore blood glucose levels, increasing glucose uptake in SKM via GLUT4 translocation to the plasma membrane and promoting lipid oxidation-inhibiting fatty acid synthesis (Janzen et al., 2018).

The association of t-miRNAs and EV-miRNAs with some of these myokines and, more specifically, the correlation of t-miR-17-5p, t-miR-29a-3p, and t-miR-885-5p with FABP3, of t-miR-532-3p with OSM, and of EV-miR-33a-5p with SPARC supported our hypothesis about a potential role for the identified t-miRNAs and EV-miRNAs in the adaptive response to endurance. Moreover, based on previous studies, some of the myokines whose expression was found changed in our experimental setting were experimentally defined as regulated by t- and EV-modulated miRNAs as described for BDNF (Varendi et al., 2014), FSTL-1, and SPARC (Galimov et al., 2015).

Of particular interest is the regulation of SPARC by hsa-miR-29a-3p, the only miRNA that, in our experimental setting, was found to be differently modulated in t- and EV-miRNA fractions. In aged muscle, it has been demonstrated that the fibroblast grow factor 2 (FGF-2) signaling pathway promotes mir-29a-3p expression and consequent SPARC reduction (Mathes et al., 2021). SPARC plays an important regulatory role in bone metabolism: it has been demonstrated that knock-out mice for osteonectin develop osteopenia and have low bone quality (Delany et al., 2000). In studies by Kapinas et al. and James et al., it has been shown that SPARC is a direct target of mir-29a in osteoblast-like MC3T3-E1 murine cell line (Kapinas et al., 2009; James et al., 2014). MiR-29 inhibition was also associated with reduced osteoclast activity (Shin et al., 2021). As previous detailed, miR-29a-3p was up-regulated in our study in t-miRNA fraction while it was down regulated in EV-miRNA fraction. Based on this evidence, the regulation of the miR-29a-SPARC axis, with miR-29a-3p decreasing in EV fraction and consequently increasing in SPARC serum level, could represent a mechanism to maintain bone and muscle homeostasis following extreme exercise. However, further *in vitro* studies are needed to verify this hypothesized mechanism.

In conclusion, this study provides an example of the possible systemic effects of acute strenuous endurance exercise. Our results highlight that such an activity induces changes in the circulating miRNA signature, thus altering cellular and tissue homeostasis to promote the exercise-induced adaptive response. This alteration results from both a passive release of miRNAs from tissues to blood, due to cell damage or increased cell membrane permeability, and miRNAs selective and controlled release into EVs for communication purposes. Although the potential biological function of the miRNAs that resulted altered by endurance exercise was speculated upon, this study only detailed the modulation of their levels, their potential effects, and their associations with known SKM-derived mediators of exercise. Therefore, further studies are needed to define the biological role of these miRNAs and to solve the question of the nature and mechanism of circulating miRNA release after exercise.

## Data Availability

The datasets presented in this study can be found in online repositories. The names of the repository/repositories and accession number(s) can be found below: https://zenodo.org/record/6376231#.YjnYPVXMIdU, 10.5281/zenodo.6376231.
