# Supplementary material for: Acute changes in free and extracellular vesicle-associated circulating miRNAs and myokine profile in professional sky-runners during the Gran Sasso d’Italia vertical run
Source: Front Mol Biosci. 2022 Aug 26;9:915080. doi: 10.3389/fmolb.2022.915080 (PMC9459384; doi:10.3389/fmolb.2022.915080)
Supplement: Supplementary file 1 [file Table1.DOCX]

Supplementary Material

# Supplementary Table 1: Analysis of Normalization Method

| **Normalization Method** | **t-miRNA** | **EV-miRNA** | **tot** |
| --- | --- | --- | --- |
| **Global Mean** | 62,53 | 72,79 | **67,66** |
| **stable miRNAs average** | 71,28 | 83,69 | 77,48 |
| **hsa-miR-151a-5p** | 63,48 | 77,54 | 70,51 |
| **hsa-miR-30d-5p** | 63,56 | 80,44 | 72 |
| **hsa-miR-361-5p** | 67,88 | 72,62 | 70,25 |
| **hsa-miR-23b-3p** | 68,83 | 72,02 | 70,42 |
| **hsa-miR-320d** | 64,69 | 73,27 | 68,98 |
| **hsa-miR-30c-5p** | 67,97 | 78,98 | 73,47 |
| **hsa-miR-23a-3p** | 67,23 | 78,38 | 72,8 |
| **hsa-miR-425-5p** | 76,29 | 76,72 | 76,5 |

Coefficient of variation analysis after normalization on Global mean, the mean of the most stable miRNAS (hsa-miR-151a-5p, hsa-miR-30d-5p, hsa-miR-361-5p, hsa-miR-23b-3p, hsa-miR-320d, hsa-miR-30c-5p, hsa-miR-23a-3p, hsa-miR-126-3p, hsa-miR-25-3p, hsa-miR-423-5p, hsa-miR-223-3p, hsa-miR-320c, hsa-miR-155-5p, hsa-miR-425-5p, hsa-miR-197-3p, hsa-miR-26b-5p, hsa-miR-199a-3p, hsa-miR-320a, hsa-miR-130a-3p, hsa-miR-320b, hsa-miR-342-3p, hsa-miR-28-3p, hsa-miR-146a-5p, hsa-miR-374b-5p, hsa-miR-146b-5p, hsa-miR-21-5p, hsa-miR-584-5p, hsa-miR-2110, hsa-miR-382-5p, hsa-miR-106a-5p, hsa-miR-20a-5p, hsa-miR-451a, hsa-miR-15b-3p, hsa-miR-324-3p, hsa-miR-409-3p, hsa-miR-192-5p, hsa-miR-125a-5p, hsa-miR-486-5p, hsa-miR-185-5p, hsa-miR-877-5p, hsa-miR-29c-3p, hsa-let7b-5p, hsa-miR-150-5p, hsa-miR-484, hsa-miR-1260a, hsa-let7g-5p, hsa-miR-140-3p, hsa-miR-126-5p) ranked by NormFinder (considering the most stable of each cluster), the first seven most stable miRNAs ranked by NormFinder (hsa-miR-151a-5p, hsa-miR-30d-5p, hsa-miR-361-5p, hsa-miR-23b-3p, hsa-miR-320d, hsa-miR-30c-5p, hsa-miR-23a-3p), and hsa-miR-425-5p (one of the most stable in serum and plasma, recommended by Exiqon) have been tested as normalizers.
