# Supplementary material for: Acute changes in free and extracellular vesicle-associated circulating miRNAs and myokine profile in professional sky-runners during the Gran Sasso d’Italia vertical run
Source: Front Mol Biosci. 2022 Aug 26;9:915080. doi: 10.3389/fmolb.2022.915080 (PMC9459384; doi:10.3389/fmolb.2022.915080)
Supplement: Supplementary file 4 [file Table2.docx]

Supplementary Material

# Supplementary Table 2: Fold-change of t-miRNAs and EV-miRNAs

| ***miRNA*** | ***fold-change*** | |
| --- | --- | --- |
|  | ***t-miRNA*** | ***EV-miRNA*** |
| hsa-let-7a-5p | 4.74 | 1.11 |
| hsa-let-7b-3p | **32.00** | 1.20 |
| hsa-let-7b-5p | 2.37 | 1.77 |
| hsa-let-7c-5p | 0.85 | 0.97 |
| hsa-let-7d-3p | 2.37 | 1.75 |
| hsa-let-7d-5p | 0.54 | 0.70 |
| hsa-let-7e-5p | 1.17 | 1.21 |
| hsa-let-7f-5p | 1.19 | 1.59 |
| hsa-let-7g-5p | 1.87 | 0.70 |
| hsa-let-7i-5p | 2.97 | 1.40 |
| hsa-miR-100-5p | 0.63 | 1.00 |
| hsa-miR-101-3p | 1.17 | 2.48 |
| hsa-miR-103a-3p | 1.20 | 0.61 |
| hsa-miR-106a-5p | 1.88 | 0.55 |
| hsa-miR-106b-3p | **32.00** | 0.58 |
| hsa-miR-106b-5p | 0.87 | 0.62 |
| hsa-miR-107 | 0.92 | 0.87 |
| hsa-miR-10b-5p | **32.00** | 2.72 |
| hsa-miR-122-5p | 3.04 | 1.99 |
| hsa-miR-125a-5p | 0.73 | 0.55 |
| hsa-miR-125b-5p | 0.79 | 2.66 |
| hsa-miR-1260a | 0.68 | 2.45 |
| hsa-miR-126-3p | 2.38 | 0.70 |
| hsa-miR-126-5p | 1.88 | 0.88 |
| hsa-miR-127-3p | 0.65 | **0.02** |
| hsa-miR-128-3p | **32.00** | 1.12 |
| hsa-miR-130a-3p | 0.69 | 0.77 |
| hsa-miR-130b-3p | 0.30 | 0.96 |
| hsa-miR-132-3p | 0.69 | 1.94 |
| hsa-miR-133a-3p | 1.00 | **32.00** |
| hsa-miR-133b | **0.02** | 1.30 |
| hsa-miR-136-3p | 1.00 | **0.03** |
| hsa-miR-136-5p | 0.37 | **53.48** |
| hsa-miR-139-5p | 0.94 | 1.73 |
| hsa-miR-1-3p | 0.40 | **0.02** |
| hsa-miR-140-3p | 2.16 | 0.61 |
| hsa-miR-140-5p | 0.98 | 0.78 |
| hsa-miR-141-3p | 1.00 | **32.00** |
| hsa-miR-142-3p | 1.89 | 1.08 |
| hsa-miR-142-5p | 0.42 | 0.99 |
| hsa-miR-143-3p | **32.97** | 2.26 |
| hsa-miR-144-3p | 1.50 | 2.54 |
| hsa-miR-144-5p | 0.35 | 0.60 |
| hsa-miR-145-5p | 0.74 | 1.95 |
| hsa-miR-146a-5p | 1.88 | 0.88 |
| hsa-miR-146b-5p | 0.65 | 1.56 |
| hsa-miR-148a-3p | 0.74 | 0.77 |
| hsa-miR-148b-3p | 0.98 | 0.88 |
| hsa-miR-150-5p | 2.35 | 2.81 |
| hsa-miR-151a-3p | 1.07 | 0.61 |
| hsa-miR-151a-5p | 1.90 | 0.70 |
| hsa-miR-152-3p | 1.17 | 1.10 |
| hsa-miR-154-5p | 0.23 | 1.57 |
| hsa-miR-155-5p | 0.68 | 1.23 |
| hsa-miR-15a-5p | 1.71 | 0.30 |
| hsa-miR-15b-3p | 0.67 | 0.77 |
| hsa-miR-15b-5p | 1.07 | 0.48 |
| hsa-miR-16-2-3p | 0.79 | 0.76 |
| hsa-miR-16-5p | 2.36 | 1.11 |
| hsa-miR-17-5p | **0.12** | 0.37 |
| hsa-miR-181a-5p | 0.63 | 1.12 |
| hsa-miR-185-5p | 2.38 | 0.88 |
| hsa-miR-186-5p | 0.63 | 1.24 |
| hsa-miR-18a-5p | 0.67 | 1.27 |
| hsa-miR-18b-5p | 1.59 | 0.87 |
| hsa-miR-191-5p | 1.48 | 0.44 |
| hsa-miR-192-5p | 2.37 | 1.11 |
| hsa-miR-193a-5p | 0.66 | 1.37 |
| hsa-miR-194-5p | 0.68 | 0.97 |
| hsa-miR-195-5p | **32.00** | 1.23 |
| hsa-miR-197-3p | 1.18 | 1.11 |
| hsa-miR-199a-3p | 1.47 | 0.71 |
| hsa-miR-199a-5p | 0.98 | 0.83 |
| hsa-miR-19a-3p | 0.94 | 0.44 |
| hsa-miR-19b-3p | 1.18 | 0.70 |
| hsa-miR-200a-3p | **32.00** | **57.65** |
| hsa-miR-200c-3p | 0.48 | 1.25 |
| hsa-miR-205-5p | **0.02** | **32.00** |
| hsa-miR-208a-3p | / | / |
| hsa-miR-20a-5p | 2.34 | 0.70 |
| hsa-miR-20b-5p | 0.43 | 1.27 |
| hsa-miR-210-3p | **32.00** | 1.29 |
| hsa-miR-2110 | 0.69 | 1.23 |
| hsa-miR-215-5p | 1.18 | 0.70 |
| hsa-miR-21-5p | 1.35 | 0.98 |
| hsa-miR-221-3p | 1.88 | 0.78 |
| hsa-miR-222-3p | 1.49 | 2.19 |
| hsa-miR-223-3p | 1.88 | 0.88 |
| hsa-miR-223-5p | 0.42 | 1.35 |
| hsa-miR-22-3p | 0.94 | 0.43 |
| hsa-miR-22-5p | 0.41 | **0.02** |
| hsa-miR-23a-3p | 1.88 | 0.69 |
| hsa-miR-23b-3p | 1.36 | 0.97 |
| hsa-miR-24-3p | 1.90 | 1.77 |
| hsa-miR-25-3p | 2.98 | 1.10 |
| hsa-miR-26a-5p | 1.87 | 0.44 |
| hsa-miR-26b-5p | 1.49 | 1.11 |
| hsa-miR-27a-3p | 1.08 | 0.38 |
| hsa-miR-27b-3p | 1.88 | 0.69 |
| hsa-miR-28-3p | 0.52 | 1.21 |
| hsa-miR-28-5p | 0.59 | 2.56 |
| hsa-miR-29a-3p | **32.00** | 0.92 |
| hsa-miR-29b-3p | 0.43 | 2.03 |
| hsa-miR-29c-3p | 0.54 | 0.77 |
| hsa-miR-301a-3p | 0.95 | 2.37 |
| hsa-miR-30a-5p | **32.00** | 0.87 |
| hsa-miR-30b-5p | 1.18 | 2.79 |
| hsa-miR-30c-5p | 1.86 | 0.70 |
| hsa-miR-30d-5p | 1.87 | 0.70 |
| hsa-miR-30e-3p | 0.66 | 1.26 |
| hsa-miR-30e-5p | 2.36 | 0.56 |
| hsa-miR-320a | 1.49 | 1.11 |
| hsa-miR-320b | 1.49 | 1.11 |
| hsa-miR-320c | 1.07 | 0.98 |
| hsa-miR-320d | 2.38 | 1.11 |
| hsa-miR-324-3p | 0.55 | 0.77 |
| hsa-miR-324-5p | 0.61 | 0.87 |
| hsa-miR-32-5p | 0.48 | 1.21 |
| hsa-miR-326 | 0.63 | **0.03** |
| hsa-miR-328-3p | 0.52 | 0.77 |
| hsa-miR-331-3p | 0.76 | 0.50 |
| hsa-miR-335-3p | 0.86 | 1.19 |
| hsa-miR-335-5p | **32.00** | 0.93 |
| hsa-miR-338-3p | 1.00 | 0.94 |
| hsa-miR-339-3p | 0.71 | 3.57 |
| hsa-miR-339-5p | 0.58 | 0.84 |
| hsa-miR-33a-5p | **32.00** | **0.02** |
| hsa-miR-342-3p | 1.87 | 1.39 |
| hsa-miR-34a-5p | **32.00** | **0.02** |
| hsa-miR-361-5p | 2.78 | 0.97 |
| hsa-miR-362-3p | **32.00** | 2.58 |
| hsa-miR-363-3p | 0.94 | 0.98 |
| hsa-miR-365a-3p | / | / |
| hsa-miR-374a-5p | 0.75 | 0.60 |
| hsa-miR-374b-5p | 0.93 | 1.39 |
| hsa-miR-375 | 1.45 | 1.25 |
| hsa-miR-376a-3p | 0.34 | 0.71 |
| hsa-miR-376c-3p | 0.47 | 0.49 |
| hsa-miR-378a-3p | 0.30 | 1.99 |
| hsa-miR-382-5p | 0.67 | 0.61 |
| hsa-miR-409-3p | 0.54 | 1.56 |
| hsa-miR-421 | 0.59 | 1.08 |
| hsa-miR-423-3p | 0.66 | 0.43 |
| hsa-miR-423-5p | 1.88 | 1.11 |
| hsa-miR-424-5p | **32.00** | 1.23 |
| hsa-miR-425-3p | 0.86 | 0.77 |
| hsa-miR-425-5p | 1.09 | 0.61 |
| hsa-miR-451a | 2.36 | 0.88 |
| hsa-miR-454-3p | 0.86 | 1.09 |
| hsa-miR-483-5p | 0.54 | 0.74 |
| hsa-miR-484 | 1.87 | 0.69 |
| hsa-miR-485-3p | 0.52 | 2.35 |
| hsa-miR-486-5p | 2.37 | 1.39 |
| hsa-miR-495-3p | 0.40 | 1.25 |
| hsa-miR-497-5p | / | / |
| hsa-miR-501-3p | **32.00** | **0.02** |
| hsa-miR-502-3p | 0.46 | 1.24 |
| hsa-miR-505-3p | 0.66 | 1.78 |
| hsa-miR-532-3p | **32.00** | 1.25 |
| hsa-miR-532-5p | 0.50 | 1.23 |
| hsa-miR-543 | **32.00** | 1.28 |
| hsa-miR-574-3p | 0.85 | 0.97 |
| hsa-miR-584-5p | 0.67 | 0.62 |
| hsa-miR-590-5p | 1.89 | 1.97 |
| hsa-miR-629-5p | 0.59 | 1.94 |
| hsa-miR-652-3p | 1.15 | 0.61 |
| hsa-miR-660-5p | 0.58 | 0.60 |
| hsa-miR-7-1-3p | 0.67 | **0.03** |
| hsa-miR-7-5p | 0.42 | 1.16 |
| hsa-miR-766-3p | 1.10 | 0.68 |
| hsa-miR-874-3p | 0.40 | **0.02** |
| hsa-miR-877-5p | 1.09 | 0.97 |
| hsa-miR-885-5p | 0.34 | **0.02** |
| hsa-miR-92a-3p | 1.35 | 0.98 |
| hsa-miR-92b-3p | 0.70 | 1.30 |
| hsa-miR-93-3p | 0.74 | 1.10 |
| hsa-miR-93-5p | 2.37 | 0.69 |
| hsa-miR-99a-5p | 1.02 | 1.32 |
| hsa-miR-99b-5p | 1.02 | 0.92 |
