# Supplementary material for: Acute changes in free and extracellular vesicle-associated circulating miRNAs and myokine profile in professional sky-runners during the Gran Sasso d’Italia vertical run
Source: Front Mol Biosci. 2022 Aug 26;9:915080. doi: 10.3389/fmolb.2022.915080 (PMC9459384; doi:10.3389/fmolb.2022.915080)
Supplement: Supplementary file 6 [file Table3.docx]

Supplementary Material

# Supplementary Table 3: Fold-Change of validated t-miRNAs and EV-miRNAs

| ***modulated miRNAs*** | ***t-miRNA*** | | | ***EV-miRNA*** | | |
| --- | --- | --- | --- | --- | --- | --- |
|  | ***fold-change*** | ***SD*** | ***p-value*** | ***fold-change*** | ***SD*** | ***p-value*** |
| hsa-let-7b-3p | 0.85 | 0.55 | 0.512 | 0.70 | 0.21 | 0.191 |
| hsa-miR-1-3p | 1.07 | 0.53 | 0.694 | 0.49 | 0.38 | **0.004** |
| hsa-miR-106b-3p | 0.92 | 0.29 | 0.589 | 1.22 | 0.89 | 0.513 |
| hsa-miR-10b-5p | 1.64 | 0.64 | **0.003** | 1.38 | 0.69 | 0.115 |
| hsa-miR-127-3p | 0.98 | 0.51 | 0.893 | 1.88 | 1.63 | 0.068 |
| hsa-miR-128-3p | 0.88 | 0.33 | 0.355 | 0.96 | 0.29 | 0.655 |
| hsa-miR-133a-3p | 0.70 | 0.47 | 0.295 | 0.87 | 0.82 | 0.776 |
| hsa-miR-133b-3p | 0.86 | 0.69 | 0.497 | 0.89 | 0.75 | 0.612 |
| hsa-miR-136-3p | 1.06 | 1.12 | 0.931 | 0.80 | 0.68 | 0.298 |
| hsa-miR-136-5p | 0.73 | 0.35 | 0.165 | 0.85 | 0.48 | 0.072 |
| hsa-miR-141-3p | 1.38 | 0.88 | 0.267 | 1.12 | 1.27 | 0.746 |
| hsa-miR-143-3p | 1.29 | 0.56 | 0.232 | 2.07 | 0.61 | **0.001** |
| hsa-miR-17-5p | 0.93 | 0.16 | 0.543 | 1.27 | 0.34 | **0.013** |
| hsa-miR-195-5p | 1.76 | 0.60 | **0.035** | 1.44 | 0.91 | 0.095 |
| hsa-miR-200a-3p | / | / | / | / | / | / |
| hsa-miR-205-5p | 1.41 | 0.90 | 0.150 | 1.02 | 0.75 | 0.947 |
| hsa-miR-210-3p | 0.95 | 0.33 | 0.623 | 1.08 | 0.61 | 0.676 |
| hsa-miR-22-5p | 0.72 | 0.42 | 0.239 | 0.92 | 0.21 | 0.675 |
| hsa-miR-29a-3p | 1.61 | 0.63 | **0.024** | 0.65 | 0.24 | **0.018** |
| hsa-miR-30a-5p | 1.25 | 0.47 | 0.161 | 1.42 | 0.80 | 0.112 |
| hsa-miR-326 | 0.71 | 0.17 | **0.038** | 0.87 | 0.89 | 0.353 |
| hsa-miR-335-5p | 0.80 | 0.34 | 0.140 | 0.94 | 0.38 | 0.661 |
| hsa-miR-33a-5p | 0.39 | 0.35 | **0.039** | 0.39 | 0.48 | 0.088 |
| hsa-miR-34a-5p | 0.85 | 0.71 | 0.725 | 3.21 | 8.29 | 0.336 |
| hsa-miR-362-3p | 0.78 | 0.60 | 0.529 | 1.11 | 0.37 | 0.377 |
| hsa-miR-424-5p | 0.75 | 0.17 | 0.136 | 0.64 | 0.34 | **>0.001** |
| hsa-miR-501-3p | 1.05 | 0.75 | 0.837 | 1.20 | 1.34 | 0.777 |
| hsa-miR-532-3p | 1.39 | 0.38 | **0.008** | 1.49 | 0.39 | **0.004** |
| hsa-miR-543 | 0.79 | 0.52 | 0.125 | 0.78 | 0.61 | 0.335 |
| hsa-miR-7-1-3p | 0.93 | 0.40 | 0.752 | 1.58 | 0.71 | 0.105 |
| hsa-miR-874-3p | 1.43 | 0.36 | 0.134 | 1.95 | 0.64 | **0.004** |
| hsa-miR-885-5p | 1.98 | 1.30 | **0.003** | 9.57 | 10.17 | **0.023** |

Fold-change of t-miRNA and EV-miRNA expression between pre- and post-race plasma samples. Difference of each miRNA expression level between the two groups was analyzed trough paired T-test. P < 0.05 was considered statistically significant (in bold).
